# Double Boosting Strategy for Low-Iodine-Dose Dual-Source DECT Follow-Up CT After Intervention with Raw DICOM-Level Deep Learning Iodine Boosting and Low-keV Dual-Energy-Derived Images

**DOI:** 10.3390/tomography12040056

**Published:** 2026-04-13

**Authors:** Tae Young Lee, Jong Hwa Lee, Hoonsub So, Ho Min Jang

**Affiliations:** 1 Department of Radiology, Ulsan University Hospital, Ulsan 44033, Republic of Korea; mromega@naver.com (T.Y.L.); dc6625@naver.com (H.M.J.); 2 Department of Radiology, University of Ulsan College of Medicine, Seoul 05505, Republic of Korea; 3 Division of Gastroenterology, Department of Internal Medicine, Ulsan University Hospital, Ulsan 44033, Republic of Korea; hoon3112@uuh.ulsan.kr; 4 Division of Gastroenterology, Department of Internal Medicine, University of Ulsan College of Medicine, Seoul 05505, Republic of Korea

**Keywords:** dual-source dual-energy CT, virtual monoenergetic imaging, deep learning reconstruction, low-iodine-dose, iodine material density image

## Abstract

**Background/Objectives**: We aim to evaluate whether digital imaging and communications in medicine (DICOM)-level deep learning-based iodine-boosting applied to dual-source dual-energy computed tomography (DECT) source DICOM improves image quality in low-iodine-dose abdominal DECT in adults undergoing post-procedure follow-up computed tomography (CT). **Methods**: This retrospective study included 43 adults (April–September 2025) who underwent dynamic dual-source DECT using a low-iodine protocol. Three CT reconstructions were compared: mixed images, conventional 50-keV virtual monoenergetic images (VMIs), and 50-keV VMIs generated after applying DICOM-based deep learning iodine-boosting/denoising to the tube-specific dual-energy source DICOM series prior to VMI/iodine-map reconstruction (deep learning-based reconstruction [DLR]-VMI). Iodine material density (IMD) images were compared between the conventional and DLR-processed datasets. Quantitative attenuation and signal-to-noise ratio (SNR) were assessed using paired and repeated-measures tests. Image quality was scored by two readers using a five-point Likert scale. **Results**: Attenuation varied across CT reconstructions for all regions of interest in both phases (all overall *p* < 0.001). Liver attenuation increased from 94.9 ± 22.0 Hounsfield units (HU) (VMI) to 114.5 ± 34.6 HU (DLR-VMI) during the arterial phase and from 127.6 ± 25.6 HU to 166.6 ± 39.9 HU during the portal venous phase (both *p* < 0.001). Liver SNR improved with DLR-VMI compared to VMI (arterial: 9.11 ± 3.62 vs. 6.06 ± 1.90; portal: 12.74 ± 3.56 vs. 7.90 ± 1.82; both *p* < 0.001). On IMD images, DLR increased HU-equivalent values and liver SNR (arterial: 5.20 ± 2.89 vs. 2.61 ± 1.39; portal: 9.22 ± 2.81 vs. 4.48 ± 1.28; both *p* < 0.001). Qualitatively, DLR-VMI yielded the highest overall image-quality scores for both reviewers in both phases (Reviewer 1, arterial/portal: 4 (4–5)/5 (4–5); Reviewer 2, arterial/portal: 4 (3–4)/4 (4–4)). DLR also improved the overall image quality of IMD images for both reviewers (all *p* < 0.001). **Conclusions**: Raw DICOM-level iodine-boosting DLR applied to dual-source DECT-source DICOM enabled enhanced image quality and improved quantitative and qualitative metrics in low-iodine-dose abdominal DECT.

## Simple Summary

Patients who need repeated computed tomography (CT) after interventions may benefit from less iodinated contrast, but organ and vessel contrast can suffer. We tested a “double boosting” approach for low-iodine-dose abdominal dual-energy CT after endoscopic ultrasound (EUS)-guided drainage. First, an artificial intelligence (AI) method was applied to the original CT data to enhance iodine signal and reduce noise. Second, dual-energy low-keV images were used for additional contrast gain. Compared with standard reconstructions, the combined approach produced clearer organs and vessels and better visibility, while keeping the iodine dose low.

## 1. Introduction

Endoscopic ultrasound (EUS)-guided transmural drainage is widely used to manage pancreatic fluid collection and has emerged as an important therapeutic option for select patients with biliary obstruction or acute cholecystitis, offering high clinical success rates and lower invasiveness than surgical or percutaneous approaches [1,2]. Despite its favorable safety profile, postprocedural complications, such as stent migration, bleeding, perforation, or secondary infection, can occur, necessitating timely and accurate radiological assessment [2,3]. Multidetector computed tomography (CT) is the primary modality for this evaluation; however, the requirement for repeated examinations around the procedure and the frequent presence of renal dysfunction in this patient population raise concerns regarding kidney safety with iodinated contrast media [4]. Consequently, there is a growing clinical demand for CT protocols that minimize iodine load without compromising diagnostic image quality.

Dual-energy CT (DECT) provides a compelling solution for low-iodine imaging by generating virtual monoenergetic images (VMIs) [5,6,7]. VMIs reconstructed at low energy levels are located closer to the k-edge of iodine (33.2 keV), substantially boosting iodine attenuation and compensating for reduced-contrast media volume. However, a major limitation of low-keV reconstruction is excessive image noise, which frequently degrades image quality and obscures fine anatomical details, potentially limiting diagnostic confidence in complex postprocedural evaluations [8].

To address this limitation, deep learning-based reconstruction (DLR) techniques have been increasingly utilized. Although DLR algorithms are primarily designed for noise reduction and contrast enhancement in standard CT imaging [9], this study proposes the application of a Digital Imaging and Communications in Medicine (DICOM)-based DLR algorithm to dual-energy source images. Applying a general-purpose DLR algorithm to the dual-energy domain preserves the underlying spectral information, thereby enabling the subsequent generation of various DLR-enhanced dual-energy derivative images such as VMIs and iodine material density (IMD) images. By combining this DLR-driven processing with low-keV VMI generation, we aimed to achieve a synergistic effect by utilizing DLR for denoising and iodine boosting while leveraging the inherent spectral contrast advantages of low-keV imaging [10].

## 2. Materials and Methods

### 2.1. Study Design and Population

At our institution, the default immediate follow-up CT after EUS-guided drainage had traditionally been non-contrast CT. However, the referring clinical teams requested contrast-enhanced follow-up imaging because non-contrast CT was often insufficient for evaluating stent position and the relevant post-procedural anatomy. In response to this clinical need, contrast-enhanced dual-energy CT was introduced for this specific follow-up setting. Based on preliminary internal clinical experience suggesting that dual-energy imaging combined with iodine-boosting post-processing could provide substantial enhancement for anatomic assessment, a reduced-iodine protocol using approximately half of the routine contrast dose was adopted in routine practice to minimize contrast burden while preserving adequate visualization of intervention-related anatomy. The present study retrospectively analyzed examinations acquired after implementation of this clinical protocol. Institutional Review Board approval was obtained, and the requirement for informed consent was waived (IRB code: UUH IRB 2025-11-035; approved on 1 December 2025). To evaluate complications following EUS-guided drainage, we included 43 adult patients (≥20 years) who underwent dynamic dual-energy abdominal CT imaging using a low-iodine-dose protocol on a SOMATOM Force scanner at Ulsan University Hospital between April and September 2025. The exclusion criteria were as follows: (1) missing required phase images in the CT protocol and (2) failure of appropriate contrast administration.

### 2.2. Image Acquisition

All CT examinations were performed using a third-generation dual-source, dual-energy CT scanner (SOMATOM Force, Siemens Healthineers, Erlangen, Germany). Dual-energy contrast-enhanced CT was acquired in dual-source mode using Tube A at 90 kVp and Tube B at Sn150 kVp, whereas non-contrast images were acquired at 120 kVp in single-energy mode. Iobitridol 350 mgI/mL (Xenetix 350 Guerbet, Villepinte, France) was administered at 0.71 mL/kg (maximum 150 mL) at a rate of 1.5 mL/s, followed by an 18-mL saline flush, corresponding to an iodine dose of 248.5 mgI/kg (350 mgI/mL × 0.71 mL/kg). Bolus tracking was performed with the region of interest (ROI) placed in the descending aorta at the diaphragmatic level, and arterial-phase images were obtained 15 s after reaching a threshold of 100 Hounsfield units (HU). Portal venous phase images were acquired using a fixed 45-s delay relative to arterial acquisition. Detailed acquisition parameters are provided in Supplementary Method S1.

### 2.3. Image Reconstruction

Three CT image sets were compared: mixed images, conventional 50-keV VMIs, and DLR-VMIs, defined as 50-keV VMIs generated after DICOM-based iodine boosting using a deep learning algorithm (ClariACE, v1.1.1, ClariPi, Seoul, Republic of Korea) applied at the raw dual-energy DICOM level. For deep learning processing, the tube-specific dual-energy DICOM series (Tube A and Tube B) were imported and processed using deep learning-based denoising and iodine boosting. The resulting tube-specific series was exported in dual-energy DICOM format and subsequently used for VMI generation and IMD image reconstruction. Conventional and DLR-processed IMD images were compared. Virtual non-contrast images were generated using the vendor workstation using a preset virtual non-contrast (VNC) algorithm based on three-material decomposition from dual-energy data. Additional reconstruction details are provided in Supplementary Method S2.

### 2.4. Quantitative Analysis

Quantitative ROI measurements were performed on individual slices. Circular ROIs >200 mm^2^ were used whenever feasible and a freehand ROI was used when a circular ROI was not feasible. ROIs were positioned within the normal liver parenchyma to avoid vessels, normal pancreatic parenchyma, renal cortex, homogeneous splenic parenchyma, and paraspinal muscle. Vascular ROIs were placed in the aorta and intrahepatic portal vein bifurcation, targeting first-order branches. ROI measurements were performed by a single abdominal radiologist with 8 years of experience, without blinding the reconstruction type during ROI placement.

### 2.5. Qualitative Image Analysis

Qualitative analysis was independently performed by a fellowship-trained abdominal radiologist (Reader 1, with 8 years of post-fellowship experience) and a radiology resident with 2 years of training experience (Reader 2). Both readers independently reviewed the mixed images, conventional 50-keV VMI, DLR 50-keV VMI, conventional IMD images, and DLR IMD images. The readers were blinded to the scan and reconstruction parameters and clinical information. No consensus reading was performed, and all image sets were scored independently by each reviewer. To minimize recall bias, image sets were presented in a random order and re-evaluated 3 months after the initial interpretation. Readers evaluated the arterial and portal venous phase image quality, assessing noise, contrast, texture, sharpness, artifacts, lesion visibility, and structure conspicuity in the liver, pancreas, and kidney, as well as upper abdominal vascular conspicuity and overall image quality. Higher scores corresponded to lower noise levels, higher contrast, more natural texture, greater sharpness, and improved overall image quality (Supplementary Table S1).

### 2.6. Radiation Dose

CT dose index volume and dose length product were extracted from the dose reports. Size-specific dose estimation (SSDE) was calculated by multiplying the CT dose index volume by a conversion factor derived from the anteroposterior and lateral diameters measured at the right renal vein level (Supplementary Figure S1).

### 2.7. Statistical Analysis

Continuous variables are presented as mean ± standard deviation, and Likert scale qualitative scores are summarized as medians [interquartile ranges]. All comparisons were performed using the same examinations (cases). CT metrics (HU and signal-to-noise ratio [SNR]) were evaluated by comparing mixed images, conventional 50-keV VMI, and DLR-VMI using repeated-measures analysis of variance, followed by Bonferroni-adjusted paired *t*-tests for post-hoc pairwise comparisons. For iodine-map metrics (HU-equivalent attenuation and liver SNR), conventional reconstructions and DLRs were compared using paired *t*-tests. Equivalence of VNC attenuation between conventional reconstructions and DLRs was assessed using two one-sided tests with a fixed equivalence margin of ±10 HU. Qualitative scores were analyzed using the Friedman test, followed by a Bonferroni-corrected post-hoc Wilcoxon signed-rank test. Inter-reader agreement was assessed using quadratic weighted kappa. All tests were two-sided, and *p* < 0.05 was considered statistically significant. Additional statistical details are provided in Supplementary Method S3.

## 3. Results

### 3.1. Demographics

A total of 43 consecutive patients who underwent abdominal CT using a low-iodine dual-energy CT protocol for postprocedural evaluation of complications following EUS-guided internal drainage were included after excluding one patient in whom intravenous access could not be secured and the examination was performed without contrast enhancement. Detailed demographics, clinical indications, and patient histories, along with radiation exposure metrics, are summarized in Table 1 and Table 2. The mean age was 71.9 ± 14.9 years (range, 34–92), with 22 men (51.2%) and 21 women (48.8%). The mean body weight was 57.1 ± 15.7 kg (range, 28.0–121.4), and the mean body mass index was 22.09 ± 4.80 kg/m^2^.

### 3.2. Quantitative Results

**Figure 1:**
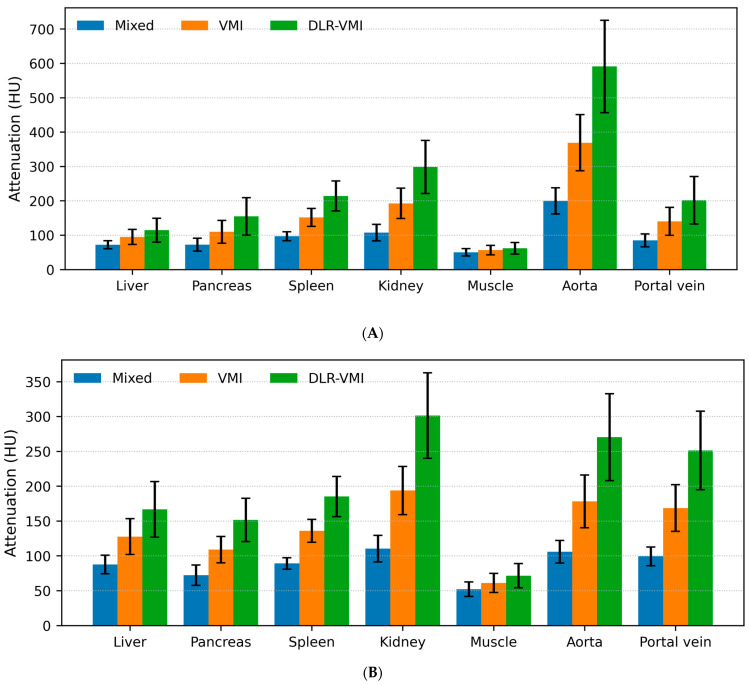
Attenuation measurements of computed tomography images by reconstruction method. Bars represent the mean attenuation, with error bars indicating standard deviations. The reconstructions included mixed images, conventional 50-keV virtual monoenergetic images (VMIs), and DLR-based 50-keV VMIs. Measurements are shown for the arterial (**A**) and portal venous (**B**) phases. Abbreviations: HU, Hounsfield unit.

Quantitative analyses were performed on 43 patients (43 examinations) using a within-examination repeated-measures design. Attenuation (HU) was compared between mixed images, conventional 50-keV VMIs, and DLR+50-keV VMIs during the arterial and portal venous phases. Significant differences in attenuation were observed across reconstructions for all ROIs of liver, pancreas, spleen, kidney, muscle, aorta and portal vein in both phases (Table 3 and Table 4; Figure 1A,B; all overall *p* < 0.001; all adjusted *p* < 0.001 for mixed vs. VMI, mixed vs. DLR-VMI, and VMI vs. DLR-VMI). For example, liver attenuation in the arterial phase was 72.0 ± 11.7 (mixed), 94.9 ± 22.0 (50-keV VMI), and 114.5 ± 34.6 (DLR+50-keV VMI), and in the portal venous phase it was 87.6 ± 13.3, 127.6 ± 25.6, and 166.6 ± 39.9, respectively. Overall, contrast-enhanced organs and vessels demonstrated increased attenuation on 50-keV VMIs compared with mixed images, whereas water and fat attenuation remained equivalent (Supplementary Table S2).

The liver SNR (liver HU divided by the standard deviation of subcutaneous fat) varied significantly across reconstructions (Table 5). In the arterial phase, liver SNR values were 7.53 ± 1.98 (mixed), 6.06 ± 1.90 (50-keV VMI), and 9.11 ± 3.62 (DLR-VMI), and in the portal venous phase, values were 8.86 ± 2.20, 7.90 ± 1.82, and 12.74 ± 3.56, respectively (all *p* < 0.001).

**Figure 2:**
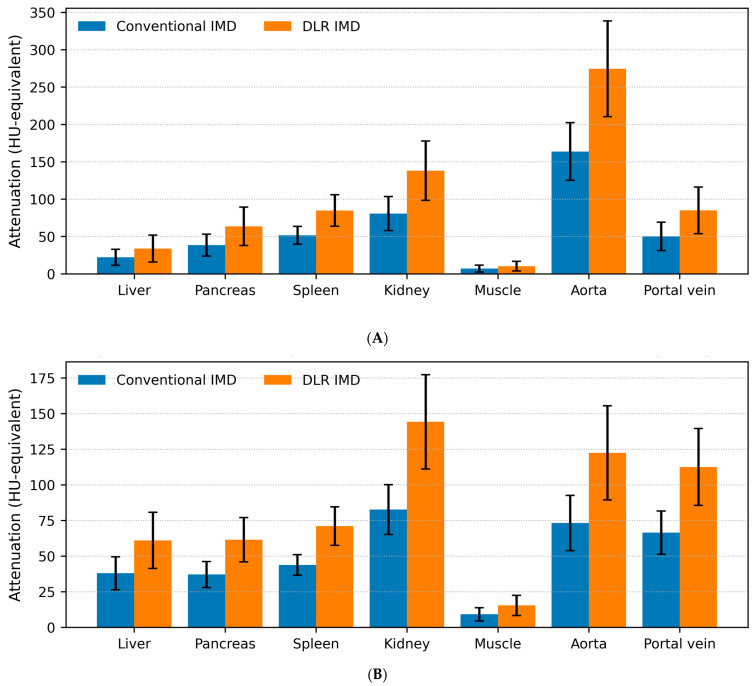
Comparison of iodine material density (IMD) image measurements obtained using conventional reconstruction and deep learning-based reconstruction (DLR) in the arterial (**A**) and portal venous phases (**B**). Bars represent the mean attenuation, with error bars indicating standard deviations. Conventional and DLR-based IMD are compared. IMD image values are reported in HU-equivalent units as provided by the vendor’s material decomposition output.

On IMD images, DLR produced higher HU-equivalent values than conventional reconstruction for most ROIs (Table 6 and Table 7; Figure 2A,B). For the liver, HU-equivalent values were 22.07 ± 10.63 vs. 33.86 ± 18.02 in the arterial phase and 37.95 ± 11.55 vs. 60.97 ± 19.67 in the portal venous phase (conventional vs. DLR). Iodine-map liver SNR increased significantly with DLR (Table 8): 2.61 ± 1.39 vs. 5.20 ± 2.89 in the arterial phase and 4.48 ± 1.28 vs. 9.22 ± 2.81 in the portal venous phase. In contrast, water and fat attenuation remained equivalent (Supplementary Table S3).

On VNC images, equivalence testing using two one-sided tests with a fixed ±10-HU margin demonstrated equivalence for the liver, pancreas, spleen, and muscle in both phases (4/7 ROIs per phase), whereas the kidney cortex and vascular ROIs (aorta and portal vein) did not meet the equivalence criterion (Table 9).

### 3.3. Qualitative Results

**Figure 3:**
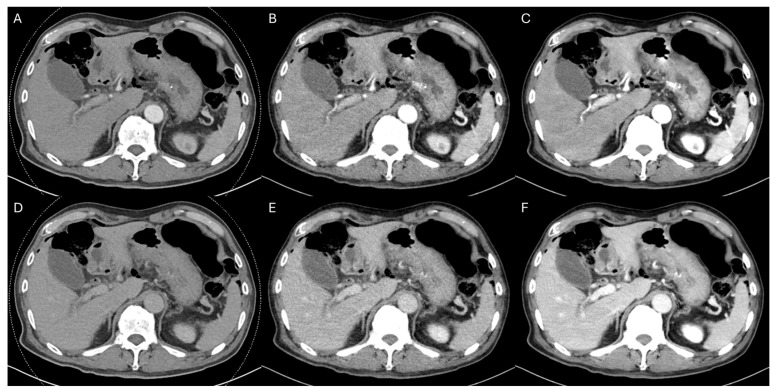
A 67-year-old man with extrahepatic cholangiocarcinoma who underwent an endoscopic ultrasound-guided hepaticogastrostomy. (**A**) Mixed image, arterial phase. (**B**) Conventional 50-keV VMI, arterial phase. (**C**) DLR-based 50-keV VMI, arterial phase. (**D**) Mixed image, portal venous phase. (**E**) Conventional 50-keV VMI, portal venous phase. (**F**) DLR-based 50-keV VMI, portal venous phase. A qualitative assessment of image quality, using a five-point Likert scale (higher scores indicate better quality), yielded the overall scores (Reviewer 1/Reviewer 2) (**A**) 3/2, (**B**) 4/3, (**C**) 5/4, (**D**) 3/3, (**E**) 4/3, and (**F**) 5/4, demonstrating higher overall image quality scores for DLR-VMI than for either the mixed image or conventional VMI in both phases. Both reviewers consistently reported improvements in noise, contrast, and sharpness during the arterial phase, and in noise and liver lesion conspicuity during the portal venous phases. Liver signal-to-noise ratios (SNRs) are provided for each panel, in the order displayed: arterial SNRs of 8.58, 6.19, and 9.17 for panels (**A**–**C**), and portal venous SNRs of 10.72, 8.44, and 14.10 for panels (**D**–**F**).

Two reviewers independently assessed the qualitative image quality of 43 examinations using a five-point Likert scale for mixed images, conventional VMI, and DLR-VMI in the arterial and portal venous phases. Overall image quality differed significantly among reconstructions in both phases for both reviewers. In the arterial phase, the median (interquartile range) overall image quality scores were 3 (3–3), 3 (3–4), and 4 (4–5) for Reviewer 1 and 3 (3–4), 3 (3–3), and 4 (3–4) for Reviewer 2 for mixed images, conventional VMI, and DLR-VMI, respectively (Friedman *p* < 0.001 for both reviewers). Bonferroni-adjusted post-hoc testing showed the following pairwise results: Reviewer 1, mixed vs. VMI *p* = 0.005, mixed vs. DLR-VMI *p* < 0.001, and VMI vs. DLR-VMI *p* < 0.001; Reviewer 2, mixed vs. VMI *p* = 1.000, mixed vs. DLR-VMI *p* = 0.001, and VMI vs. DLR-VMI *p* < 0.001. In the portal venous phase, the corresponding scores were 3 (2–3), 4 (3–4), and 5 (4–5) for Reviewer 1 and 3 (3–4), 3 (3–3), and 4 (4–4) for Reviewer 2 (Friedman *p* < 0.001 for both reviewers; Reviewer 1, all pairwise *p* < 0.001; Reviewer 2, mixed vs. VMI *p* = 1.000, mixed vs. DLR-VMI *p* < 0.001, and VMI vs. DLR-VMI *p* < 0.001) (Figure 3; Supplementary Tables S4–S7). DLR-VMI yielded the highest overall image quality in both phases for both reviewers.

Conventional and DLR IMD images were compared for qualitative image quality. For Reviewer 1, the overall image quality improved with DLR in both phases (arterial: 2 (2–3) vs. 4 (3–4), Wilcoxon *p* < 0.001; portal venous: 2 (2–3) vs. 4 (4–5), *p* < 0.001). For Reviewer 2, DLR similarly improved overall image quality (arterial: 2 (2–3) vs. 3 (3–4), *p* < 0.001; portal venous: 3 (2–3) vs. 4 (4–4), *p* < 0.001) (Supplementary Figure S2; Supplementary Tables S8–S11).

Other qualitative metrics demonstrated concordant effects of DLR for both reviewers (Supplementary Tables S4–S11). Compared with conventional VMI, DLR-VMI scored higher in both phases for noise, image contrast, liver and pancreatic lesion/structure conspicuity, vascular conspicuity, and overall image quality (Bonferroni-adjusted *p* < 0.05 for all) (Supplementary Tables S4–S7)). In the arterial phase only, DLR-VMI also received higher scores for kidney lesion/structure conspicuity than conventional VMI (Bonferroni-adjusted *p* < 0.05) (Supplementary Tables S4 and S6). Compared with mixed images, DLR-VMI scored higher in both phases for image contrast, liver/pancreas/kidney lesions or structural conspicuity, vascular conspicuity, and overall image quality (Bonferroni-adjusted *p* < 0.05 for all) (Supplementary Tables S4–S7). For IMD images, DLR processing scored higher than conventional IMD images in both phases for noise, image contrast, liver/pancreas/kidney lesions or structural conspicuity, vascular conspicuity, and overall image quality (*p* < 0.05 for all) (Supplementary Tables S8–S11). In the portal phase only, DLR IMD images also received higher sharpness scores than conventional IMD images (*p* < 0.05) (Supplementary Tables S9 and S11). In contrast, texture scores were consistently lower with DLR processing on both CT and IMD images in both phases (*p* < 0.05 for both reviewers), although the median texture score remained at 4. Furthermore, compared with mixed images, DLR-VMI received lower sharpness and artifact scores in the arterial phase (*p* < 0.05 for both reviewers), whereas the median scores for these metrics remained high (median 4) (Supplementary Tables S4 and S6). For both reviewers, DLR yielded a greater improvement in overall image quality on IMD images than on CT (VMI). The iodine-map gain exceeded the CT gain in both phases (Reviewer 1: arterial *p* < 0.001 and portal venous *p* < 0.001; Reviewer 2: arterial *p* = 0.012 and portal venous *p* = 0.011). Inter-reader agreement (quadratic weighted κw) for overall image quality was 0.54 in the arterial phase and 0.63 in the portal venous phase for mixed CT and VMIs, and 0.72 (arterial) and 0.75 (portal venous) for IMD images (Supplementary Table S12).

## 4. Discussion

In post-interventional pancreatobiliary and hepatobiliary care, contrast-enhanced CT is frequently repeated to confirm technical success and exclude complications such as hemorrhage, bile leak, abscess formation, or vascular events. In this setting, repeated iodinated contrast exposure can be clinically undesirable, particularly in older patients or those with a reduced renal reserve, and current practice guidance emphasizes individual risk stratification and the use of the minimum contrast dose required to answer clinical questions [11]. Therefore, strategies that preserve imaging quality while reducing iodine delivery have substantial clinical value. Our results support the idea that DLR can compensate for the inherent loss of enhancement and increased noise expected at low iodine doses, enabling sufficient image quality for interpretation. When repeated follow-up CT is anticipated in high-risk patients after intervention, reducing the iodine burden while maintaining high-quality, high-confidence imaging may improve the overall safety margin of longitudinal care without compromising the clinical utility of CT in detecting postprocedural complications and guiding management [11,12].

A recent study demonstrated that hepatic multiphase CT using low-concentration iodine contrast combined with low-monoenergetic imaging can achieve non-inferior image quality and lesion detectability compared with standard protocols, supporting the feasibility of iodine reduction strategies when VMI is applied [13]. Although low-keV VMI from DECT can amplify iodine attenuation, thereby increasing vascular and parenchymal enhancement, this benefit may be partially offset by higher image noise, which can undermine effective image quality and subjective interpretability [14]. Recognized technical limitations inherent to some DECT platforms, such as cross-scatter radiation in dual-source geometries, can degrade DECT image quality [14]. To address this trade-off, “noise-optimized” monoenergetic approaches have been developed and shown to improve quantitative and qualitative parameters for contrast-dependent tasks [15]. More recently, combining DLR with low-energy VMI has been reported as a practical strategy to suppress low-keV noise and improve diagnostic visibility by enhancing delineation in contrast-enhanced DECT applications [16]. Our results are concordant with these observations. DLR mitigated the noise penalty associated with low-keV VMI, allowing the iodine-boosting advantage of VMI to translate into improved image quality metrics. Furthermore, our “double-boosting” concept—pairing iodine signal boosting by low-keV VMI with noise suppression by DLR—enabled the use of approximately half of the routine contrast dose while maintaining robust enhancement and overall image quality. This represents a more aggressive level of iodine reduction than prior abdominal CT studies using DLR alone or combining low-keV VMI with DLR denoising, where acceptable or improved image quality has been reported with reduced iodine load through lower iodine concentration or weight-based iodine dose reduction in abdominal CT, and is directionally consistent with clinical evidence that deep learning-based contrast-boosting can preserve image quality under reduced-contrast conditions [9,17,18,19].

By directly boosting iodine information at the raw DICOM level, our approach enables the generation of high-quality IMD images, which function as subtraction-like images that depict iodine-specific enhancement while suppressing non-enhancing background components for the detection of subtly enhanced lesions [20,21,22]. In our study, most non-iodine-driven materials (e.g., fat and water regions on VMI/IMD images) and most organ ROIs on the VNC (liver, pancreas, spleen, muscle, fat, and water) demonstrated differences that were visually acceptable and largely within the predefined HU equivalence margin. Conversely, strongly enhancing structures (renal cortex and vascular ROIs) showed HU differences that exceeded the equivalence margin. A plausible explanation is that material decomposition and iodine quantification on DECT are subject to technical limitations, and accuracy may deteriorate depending on iodine concentration, object size, spectral separation, and system-specific factors, particularly under conditions of high iodine signal and complex beam hardening/scatter effects. In other words, when the enhancement is very strong, DECT-based iodine estimation and derived images (including the VNC) may be more susceptible to quantification bias, potentially contributing to the out-of-margin discrepancies observed in the kidney and vascular ROIs in our dataset [23]. Importantly, the DLR used in our study was not a pure denoising algorithm. Given its potential to alter CT numbers beyond noise suppression, it may interact with DECT material decomposition and influence iodine estimation and derived images, particularly in strongly enhanced structures. These limitations may pose challenges for precise quantitative material analyses.

One notable drawback of DLR in our study was the artificially smooth or “plastic-like” image texture, consistently recognized by all readers. This phenomenon, described in iterative CT reconstruction literature as an unfamiliar or blotchy/plastic appearance, can emerge when aggressive noise suppression algorithms alter the noise power spectrum and perceived texture, potentially masking subtle visual cues that radiologists unconsciously rely upon for confident interpretation [24]. Such textural alterations are clinically relevant because they may partially offset the benefits of improved contrast and reduced noise, particularly for tasks that depend on fine textural details. Although DLR is designed to preserve a more natural noise texture than conventional iterative methods, higher reconstruction strengths may still introduce increased image smoothing and subtle loss of fine detail, which can be perceived as an artificial or “plastic-like” appearance by readers [25]. In our study, texture ratings were indeed lower on DLR-processed images; however, the median texture score remained relatively high (median 4), suggesting that the degree of “plastic” appearance was noticeable but generally acceptable for clinical reading. We speculate that this mitigation may be related to the final vendor-side image processing and display pipeline applied after DLR, which could temper overtly artificial texture compared to the DLR output alone. However, this interpretation remains hypothesized and warrants confirmation in future studies that explicitly compare texture characteristics across each post-processing step.

This study has some limitations. First, its retrospective design, conducted at a single institution, and modest sample size limited its statistical power and generalizability. Second, we primarily assessed image quality endpoints (quantitative metrics and Likert-based qualitative scores) and did not evaluate whether the improved appearance translated into clinically meaningful gains in lesion detection or the diagnosis of complications following EUS-guided drainage. This is particularly relevant because drainage-related complications are relatively infrequent and heterogeneous. Analyzing results focused on rare complications in a small cohort presents a challenging task. Third, deep learning-based processing can alter CT numbers and image texture, necessitating caution when strict quantitative interpretation is required (e.g., attenuation-based thresholds, longitudinal HU tracking, or material-decomposition-derived metrics). Prior research has specifically evaluated how DLR may influence the accuracy and variability of iodine quantification in DECT, underscoring that quantitative behavior can differ according to the reconstruction method and setting. Fourth, the qualitative assessment may have been influenced by reader heterogeneity; the two readers had notably different levels of experience, and the less-experienced reader may have been more susceptible to unfamiliar DLR textures or contrast presentations, potentially influencing the subjective ratings. Fifth, our cohort consisted of post-interventional patients (following EUS-guided drainage), and factors specific to this population (postprocedural changes, indwelling devices, and short-interval repeat imaging) may limit the extrapolation to routine, non-interventional, and abdominal CT populations. Finally, we focused on quantitative and qualitative image quality metrics and did not directly assess diagnostic performance, such as lesion detectability, sensitivity/specificity, or clinical outcomes. Future studies are warranted to evaluate the diagnostic impact of this approach using reader-performance and lesion-based analyses. In addition, using the expanded cohort from our clinical workflow, we plan further retrospective studies focusing on the detection and characterization of hepatic and pancreatic lesions.

## 5. Conclusions

Our findings suggest that low-iodine-dose abdominal DECT combined with an artificial intelligence-based iodine/contrast-boosting approach and dual-energy-derived boosting (low-keV VMI and IMD images) with deep learning-based noise suppression can provide robust organ and vascular enhancement, improved lesion conspicuity, and effective noise reduction in adult patients undergoing follow-up CT after intervention. Further studies are warranted to determine whether these image quality improvements translate into clinically meaningful gains in diagnostic performance, including lesion detectability and characterization in real-world practice.

## Figures and Tables

**Table 1:** Patient demographics and clinical indications/history.

Variable	N	Value	Range
Demographics			
Number of patients	43		
Sex			
Male, n (%)	22	51.2%	
Female, n (%)	21	48.8%	
Age (years)	43	71.9 ± 14.9	34.0–92.0
Body weight (kg)	43	57.05 ± 15.71	28.00–121.40
Body mass index (kg/m^2^)	43	22.09 ± 4.80	12.78–42.01
Anteroposterior diameter (mm)	43	237.7 ± 47.5	156.0–353.0
Lateral diameter (mm)	43	282.7 ± 46.7	184.0–417.0
AP + lateral diameter (cm)	43	52.04 ± 6.62	36.70–77.00
Clinical history/indication			
Acute cholecystitis	12	27.9%	
Pancreatic cancer	11	25.6%	
Extrahepatic cholangiocarcinoma	9	20.9%	
Chronic pancreatitis	2	4.7%	
CBD stone	2	4.7%	
Others	7	16.3%	

Note: Continuous variables are presented as mean ± SD with ranges. Categorical variables are presented as n (%). Perihilar and extrahepatic cholangiocarcinoma were combined and reported as extrahepatic cholangiocarcinoma. ‘Others’ includes cholecystitis (n = 1), pancreatitis (n = 1), colon cancer (n = 1), duodenal cancer (n = 1), pancreatic neuroendocrine tumor (n = 1), ampulla of Vater cancer (n = 1), and gallbladder cancer (n = 1).

**Table 2:** Radiation exposure.

Variable	Value	Range
CTDIvol (arterial phase) (mGy)	5.53 ± 2.66	2.71–17.28
CTDIvol (portal venous phase) (mGy)	5.60 ± 2.18	2.69–15.07
DLP (arterial phase) (mGy·cm)	175.9 ± 101.9	61.7–667.3
DLP (portal venous phase) (mGy·cm)	311.6 ± 141.9	116.0–967.8
DLP (total exam) (mGy·cm)	640.6 ± 328.4	237.0–2200.0
SSDE (arterial phase) (mGy)	7.73 ± 2.77	5.22–20.98
SSDE (portal venous phase) (mGy)	7.90 ± 2.20	5.22–18.11

Note: CTDIvol, CT dose index volume; DLP, dose-length product; SSDE, size-specific dose estimate.

**Table 3:** Arterial-phase attenuation (HU) across reconstructions.

Structure/ROI	Mixed (HU)	VMI (HU)	DLR-VMI (HU)	Overall *p*	Mixed vs. VMI ^a^	Mixed vs. DLR-VMI ^a^	VMI vs. DLR-VMI ^a^
Liver	72.0 ± 11.7	94.9 ± 22.0	114.5 ± 34.6	<0.001	<0.001	<0.001	<0.001
Pancreas	72.5 ± 18.9	109.7 ± 33.0	154.6 ± 54.5	<0.001	<0.001	<0.001	<0.001
Spleen	96.7 ± 13.0	151.9 ± 26.1	214.0 ± 43.5	<0.001	<0.001	<0.001	<0.001
Kidney	107.1 ± 24.0	192.7 ± 44.0	298.5 ± 77.1	<0.001	<0.001	<0.001	<0.001
Muscle	50.1 ± 10.6	56.5 ± 13.7	61.8 ± 16.8	<0.001	<0.001	<0.001	<0.001
Aorta	199.6 ± 38.2	369.0 ± 81.8	590.9 ± 134.5	<0.001	<0.001	<0.001	<0.001
Portal vein	84.9 ± 18.6	140.1 ± 40.6	201.6 ± 69.2	<0.001	<0.001	<0.001	<0.001

Note: Data are presented as mean ± SD (HU). Spleen-related measurements excluded one examination due to prior splenectomy. ^a^ Adjusted *p*-value with Bonferroni correction for three pairwise comparisons within each row.

**Table 4:** Portal venous-phase attenuation (HU) across reconstructions.

Structure/ROI	Mixed (HU)	VMI (HU)	DLR-VMI (HU)	Overall *p*	Mixed vs. VMI ^a^	Mixed vs. DLR-VMI ^a^	VMI vs. DLR-VMI ^a^
Liver	87.6 ± 13.3	127.6 ± 25.6	166.6 ± 39.9	<0.001	<0.001	<0.001	<0.001
Pancreas	72.3 ± 14.6	108.9 ± 18.8	151.6 ± 31.0	<0.001	<0.001	<0.001	<0.001
Spleen	89.1 ± 8.2	135.8 ± 16.5	185.1 ± 28.8	<0.001	<0.001	<0.001	<0.001
Kidney	110.3 ± 19.1	193.8 ± 34.6	301.3 ± 61.4	<0.001	<0.001	<0.001	<0.001
Muscle	52.2 ± 10.4	61.1 ± 13.6	71.5 ± 17.5	<0.001	<0.001	<0.001	<0.001
Aorta	105.9 ± 16.2	178.2 ± 37.8	270.3 ± 62.4	<0.001	<0.001	<0.001	<0.001
Portal vein	99.3 ± 13.4	168.5 ± 33.6	251.2 ± 56.3	<0.001	<0.001	<0.001	<0.001

Note: Data are presented as mean ± SD (HU). Spleen-related measurements excluded one examination due to prior splenectomy. ^a^ Adjusted *p*-value with Bonferroni correction for three pairwise comparisons within each row.

**Table 5:** Liver SNR on CT across reconstructions.

Metric	Mixed	VMI	DLR-VMI	Overall *p*	Mixed vs. VMI ^a^	Mixed vs. DLR-VMI ^a^	VMI vs. DLR-VMI ^a^
Liver SNR (Arterial)	7.5 ± 2.0	6.1 ± 1.9	9.1 ± 3.6	<0.001	<0.001	<0.001	<0.001
Liver SNR (Portal venous)	8.9 ± 2.2	7.9 ± 1.8	12.7 ± 3.6	<0.001	<0.001	<0.001	<0.001

Note: Data are presented as mean ± SD. SNR was calculated as liver attenuation divided by the standard deviation (SD) of subcutaneous fat attenuation. ^a^ Adjusted *p*-value with Bonferroni correction for three pairwise comparisons within each row.

**Table 6:** HU-equivalent attenuation on IMD images in the arterial phase (conventional vs. DLR).

ROI	Conventional	DLR	Δ(DLR−Conv)	*p*
Liver	22.07 ± 10.63	33.86 ± 18.02	11.79 (9.15, 14.44)	<0.001
Pancreas	38.41 ± 14.59	63.59 ± 25.68	25.18 (21.46, 28.89)	<0.001
Spleen	51.62 ± 11.98	84.78 ± 21.03	33.17 (30.04, 36.30)	<0.001
Kidney	80.58 ± 22.76	138.10 ± 39.71	57.52 (52.02, 63.02)	<0.001
Muscle	6.90 ± 4.65	10.20 ± 6.44	3.30 (2.38, 4.22)	<0.001
Aorta	163.67 ± 38.65	274.40 ± 64.14	110.73 (101.74, 119.72)	<0.001
Portal vein	50.03 ± 18.95	84.99 ± 31.18	34.96 (30.07, 39.85)	<0.001

Note: Data are presented as mean ± SD (HU-equivalent). Δ(DLR−Conv) is the mean paired difference with 95% CI in parentheses. Spleen-related measurements excluded one examination due to prior splenectomy.

**Table 7:** HU-equivalent attenuation on IMD images in the portal venous phase (conventional vs. DLR).

ROI	Conventional	DLR	Δ(DLR−Conv)	*p*
Liver	37.95 ± 11.55	60.97 ± 19.67	23.02 (19.84, 26.19)	<0.001
Pancreas	37.03 ± 9.13	61.48 ± 15.56	24.46 (21.95, 26.96)	<0.001
Spleen	43.77 ± 7.21	71.03 ± 13.50	27.26 (24.69, 29.84)	<0.001
Kidney	82.63 ± 17.48	144.17 ± 33.12	61.54 (56.34, 66.74)	<0.001
Muscle	9.13 ± 4.64	15.36 ± 7.09	6.24 (4.99, 7.49)	<0.001
Aorta	73.15 ± 19.42	122.42 ± 33.06	49.27 (44.83, 53.72)	<0.001
Portal vein	66.41 ± 15.13	112.50 ± 26.97	46.09 (41.87, 50.30)	<0.001

Note: Data are presented as mean ± SD (HU-equivalent). Δ(DLR−Conv) is the mean paired difference with 95% CI in parentheses. Spleen-related measurements excluded one examination due to prior splenectomy.

**Table 8:** Liver SNR on IMD images (conventional vs. DLR).

	Conventional	DLR	Δ(DLR−Conv)	*p*
Liver SNR (Arterial)	2.6 ± 1.4	5.2 ± 2.9	2.59 (2.06, 3.13)	<0.001
Liver SNR (Portal venous)	4.5 ± 1.3	9.2 ± 2.8	4.75 (4.15, 5.34)	<0.001

Note: Data are presented as mean ± SD (HU-equivalent). Δ(DLR−Conv) is the mean paired difference with 95% CI in parentheses.

**Table 9:** Equivalence assessment for VNC attenuation (conventional vs. DLR; margin ±10 HU).

Phase	ROI	Margin (HU)	Bias (HU)	LOA (HU)	TOST *p*_low	TOST *p*_high	Equivalence
Arterial	Liver	10	4.29	(−4.37, 12.95)	<0.001	<0.001	Pass
Arterial	Pancreas	10	6.68	(−2.91, 16.27)	<0.001	<0.001	Pass
Arterial	Spleen	10	6.66	(−1.60, 14.92)	<0.001	<0.001	Pass
Arterial	Kidney	10	11.69	(0.06, 23.33)	<0.001	0.966	Fail
Arterial	Muscle	10	1.57	(−1.43, 4.57)	<0.001	<0.001	Pass
Arterial	Aorta	10	9.57	(−2.77, 21.90)	<0.001	0.327	Fail
Arterial	Portal vein	10	10.45	(1.21, 19.69)	<0.001	0.732	Fail
Arterial	Water	10	1.26	(−4.28, 6.80)	<0.001	<0.001	Pass
Arterial	Fat	10	0.56	(−2.23, 3.36)	<0.001	<0.001	Pass
Portal venous	Liver	10	8.46	(−0.81, 17.72)	<0.001	0.019	Pass
Portal venous	Pancreas	10	7.23	(−0.58, 15.03)	<0.001	<0.001	Pass
Portal venous	Spleen	10	7.25	(−0.44, 14.93)	<0.001	<0.001	Pass
Portal venous	Kidney	10	16.71	(4.73, 28.69)	<0.001	1.000	Fail
Portal venous	Muscle	10	2.87	(−0.56, 6.29)	<0.001	<0.001	Pass
Portal venous	Aorta	10	9.43	(−0.32, 19.18)	<0.001	0.230	Fail
Portal venous	Portal vein	10	14.98	(5.17, 24.79)	<0.001	1.000	Fail
Portal venous	Water	10	2.44	(−2.25, 7.14)	<0.001	<0.001	Pass
Portal venous	Fat	10	0.84	(0.19, 1.50)	<0.001	<0.001	Pass

Note: Bias is the mean paired difference (Conventional − DLR). LOA denotes 95% limits of agreement. Equivalence was assessed using two one-sided tests (TOST) with a margin of ±10 HU. Spleen measurements excluded one examination due to prior splenectomy. Water measurements excluded three examinations because no clean intraperitoneal fluid was available for ROI placement.

## Data Availability

Individual patient data will not be shared due to privacy concerns. Data generated or analyzed during the study are available from the corresponding author by request.

## Supplementary Materials

The following supporting information can be downloaded at https://www.mdpi.com/article/10.3390/tomography12040056/s1: Methods S1: Detailed acquisition parameters; Methods S2: Deep learning processing settings and reconstruction details; Methods S3: Additional statistical details; Figure S1: Size-specific dose estimate (SSDE) calculation in a 45-year-old man. An example of how to obtain the anteroposterior and lateral dimensions at the mid-scan image to calculate the effective diameter. Figure S2: A 77-year-old woman with pancreatic cancer, status post ERPD, ERBD, and EUS-guided cholecystoduodenostomy; Table S1: Qualitative image quality evaluation criteria; Table S2: Equivalence assessment for water and fat attenuation on VMI (VMI vs. DLR-VMI; margin ±10 HU); Table S3: Equivalence assessment for fat and water attenuation on IMD images (margin ±10 HU); Table S4: Qualitative scores on CT images (Reviewer 1)—Arterial phase; Table S5: Qualitative scores on CT images (Reviewer 1)—Portal venous phase; Table S6: Qualitative scores on CT images (Reviewer 2)—Arterial phase; Table S7: Qualitative scores on CT images (Reviewer 2)—Portal venous phase; Table S8: Qualitative scores on IMD images (Reviewer 1)—Arterial phase; Table S9: Qualitative scores on IMD images (Reviewer 1)—Portal venous phase; Table S10: Qualitative scores on IMD images (Reviewer 2)—Arterial phase; Table S11: Qualitative scores on IMD images (Reviewer 2)—Portal venous phase; Table S12: Inter-reader agreement (quadratic weighted kappa, κw).

## Abbreviations

The following abbreviations are used in this manuscript:

AI	Artificial intelligence
AP	Anteroposterior
CBD	Common bile duct
CT	Computed tomography
CTDIvol	CT dose index volume
DECT	Dual-energy computed tomography
DICOM	Digital Imaging and Communications in Medicine
DLR	Deep learning-based reconstruction
DLP	Dose-length product
EUS	Endoscopic ultrasound
HU	Hounsfield unit
IMD	Iodine material density
IRB	Institutional Review Board
ROI	Region of interest
SD	Standard deviation
SNR	Signal-to-noise ratio
SSDE	Size-specific dose estimate
TOST	Two one-sided tests
VMI	Virtual monoenergetic image
VNC	Virtual non-contrast
