# Supplementary material for: Double Boosting Strategy for Low-Iodine-Dose Dual-Source DECT Follow-Up CT After Intervention with Raw DICOM-Level Deep Learning Iodine Boosting and Low-keV Dual-Energy-Derived Images
Source: Tomography. 2026 Apr 13;12(4):56. doi: 10.3390/tomography12040056 (PMC13119852; doi:10.3390/tomography12040056)
Supplement: Supplementary file 1 [file tomography-12-00056-s001.zip › tomography-4189135-Supplementary.pdf]

Supplementary Methods

Supplementary Methods S1: Detailed acquisition parameters

Automatic exposure control with tube current modulation (CARE Dose4D, Siemens Healthineers, Forchheim, Germany) was used (CARE kV, Siemens Healthineers, Forchheim, Germany, was not used for dual-energy acquisitions), with a quality reference mAs of 210. Tube-current allocation followed the vendor-default settings (approximately 210 mAs for Tube A and 131 mAs for Tube B). The acquisition parameters included a collimation of 192×0.6 mm×2, rotation time of 0.5 s, pitch of 0.6, and craniocaudal scan direction.

Supplementary Methods S2: Deep learning processing settings and reconstruction details

The denoise model was set to "standard", the denoise strength to "standard", and the default boost strength to "boost". Image sets were defined as follows: (1) mixed images, linearly blended dual-energy images (0.7×90 kVp + 0.3×Sn150 kVp; M_0.7); (2) conventional VMIs at 50 keV generated on syngo.via, VB80 (Siemens Healthineers, Forchheim, Germany); and (3) DLR-VMI, 50-keV VMI generated after DICOM-based iodine boosting using ClariACE, version 1.1.1 (ClariPi, Seoul, Republic of Korea) applied to dual-energy source DICOM from each tube. The iodine material density images were generated using vendor-preset dual-energy material decomposition algorithms. Virtual non-contrast generation used vendor-preset three-material decomposition with the liver VNC algorithm.

Supplementary Methods S3: Additional statistical details

When medians and interquartile ranges were identical, the direction of differences was determined from paired comparisons by creating a difference variable and conducting a one-sample Wilcoxon signed-rank test against zero. For each examination, we calculated the DLR-related gain in overall image quality as the score difference between DLR and conventional reconstruction for CT (DLR-VMI minus VMI) and IMD images (DLR IMD image minus conventional IMD image) in each phase and then compared these paired gains (iodine-map gain vs. CT gain) using a paired sign test. Statistical analysis was performed using commercially available software, version 28.0 (IBM Corp., Armonk, NY, USA).

Supplementary Tables

Table S1. Qualitative image quality evaluation criteria

Parameters	Score	Scoring system
Noise	1–5	Score 1, non-diagnostic due to excessive noise<br>Score 2, substantial noise affecting diagnostic confidence<br>Score 3, diagnostically acceptable but noticeable noise<br>Score 4, mild noise with no or only mild quality decrease<br>Score 5, no perceivable noise

Contrast	1–5	Score 1, markedly insufficient enhancement similar to pre-contrast image<br>Score 2, poor contrast<br>Score 3, average contrast comparable to routine practice<br>Score 4, sufficient contrast for confident diagnosis<br>Score 5, excellent/strong contrast
Texture	1–5	Score 1, non-diagnostic (severe blotchy/pixelated or unnatural appearance)<br>Score 2, significant blotchy/pixelated appearance affecting confidence<br>Score 3, diagnostically acceptable but noticeable texture degradation/smoothing<br>Score 4, mild smoothing/blotchiness with no or mild quality decrease<br>Score 5, natural texture without blotchy/pixelated appearance
Sharpness	1–5	Score 1, non-diagnostic due to severe blurring<br>Score 2, substantial blurring reducing diagnostic confidence<br>Score 3, diagnostically acceptable with noticeable blurring<br>Score 4, good sharpness with only mild blurring<br>Score 5, excellent sharpness and edge definition
Artifacts	1–5	Score 1, severe artifacts (non-diagnostic)<br>Score 2, substantial artifacts affecting diagnostic confidence<br>Score 3, moderate artifacts but diagnostically acceptable<br>Score 4, mild artifacts with no or mild quality decrease<br>Score 5, no perceivable artifacts
Liver lesion/structure conspicuity	1–5	Score 1, non-diagnostic; lesions/structures not discernible<br>Score 2, poor conspicuity limiting diagnosis<br>Score 3, acceptable conspicuity comparable to routine practice<br>Score 4, good conspicuity with confident evaluation<br>Score 5, excellent conspicuity
Pancreas lesion/structure conspicuity	1–5	Score 1, non-diagnostic; lesions/structures not discernible<br>Score 2, poor conspicuity limiting diagnosis<br>Score 3, acceptable conspicuity comparable to routine practice<br>Score 4, good conspicuity with confident evaluation<br>Score 5, excellent conspicuity

Kidney lesion/structure conspicuity	1–5	Score 1, non-diagnostic; lesions/structures not discernible<br>Score 2, poor conspicuity limiting diagnosis<br>Score 3, acceptable conspicuity comparable to routine practice<br>Score 4, good conspicuity with confident evaluation<br>Score 5, excellent conspicuity
Vascular conspicuity	1–5	Score 1, non-diagnostic; vessels poorly visualized<br>Score 2, poor vessel delineation limiting evaluation<br>Score 3, acceptable vessel delineation comparable to routine practice<br>Score 4, good vessel delineation with confident evaluation<br>Score 5, excellent vessel delineation
Overall image quality	1–5	Score 1, non-diagnostic<br>Score 2, poor image quality limiting diagnosis<br>Score 3, fair image quality for routine clinical practice<br>Score 4, good image quality with confident evaluation<br>Score 5, excellent

Table S2. Equivalence assessment for water and fat attenuation on VMI (VMI vs DLR-VMI; margin ±10 HU)

Section	ROI	Margin (HU)	Bias (HU)	LOA (HU)	TOST p_low	TOST p_high	Equivalence
Arterial VMI (50 keV)	Water	10	-1.13	(-9.62, 7.36)	<0.001	<0.001	Pass
Arterial VMI (50 keV)	Fat	10	0.28	(-3.32, 3.89)	<0.001	<0.001	Pass
Portal venous VMI (50 keV)	Water	10	-4.36	(-20.10, 11.37)	<0.001	<0.001	Pass
Portal venous VMI (50 keV)	Fat	10	0.18	(-1.46, 1.82)	<0.001	<0.001	Pass

Note: Bias is the mean paired difference (VMI − DLR-VMI). LOA denotes Bland–Altman 95% limits of agreement. Equivalence was assessed using two one-sided tests (TOST) with a fixed ±10 HU margin. Water analyses excluded three examinations because no sufficiently clean intraperitoneal fluid was available for ROI placement.

Table S3. Equivalence assessment for fat and water attenuation on IMD images (margin ±10 HU)

Section	ROI	Margin (HU)	Bias (HU)	LOA (HU)	TOST p_low	TOST p_high	Equivalence

Arterial IMD image	Fat	10	-0.05	(-0.64, 0.54)	<0.001	<0.001	Pass
Arterial IMD image	Water	10	-1.12	(-4.93, 2.70)	<0.001	<0.001	Pass
Portal venous IMD image	Fat	10	-0.27	(-1.30, 0.77)	<0.001	<0.001	Pass
Portal venous IMD image	Water	10	-2.29	(-9.20, 4.61)	<0.001	<0.001	Pass

Note: Bias is the mean paired difference (Conventional − DLR). LOA denotes 95% limits of agreement. Equivalence was assessed using two one-sided tests (TOST) with a ±10 HU margin. Water-related analyses excluded three examinations because an adequately homogeneous water-equivalent region was not available for ROI placement.

Table S4. Qualitative scores on CT images (Reviewer 1) - Arterial phase

Criterion	Mixed	VMI	DLR-VMI	Friedman p	Kendall W	Mixed vs VMI[a]	Mixed vs DLR[a]	VMI vs DLR[a]
Noise	4 (4–5)	3 (3–3)	5 (4–5)	<0.001	0.785	<0.001	0.043	<0.001
Contrast	2 (2–3)	3 (3–4)	5 (4–5)	<0.001	0.816	<0.001	<0.001	<0.001
Texture	5 (5–5)	5 (5–5)	4 (4–4)	<0.001	0.629	0.020[b]	<0.001	<0.001
Sharpness	5 (4–5)	4 (4–4)	4 (4–5)	<0.001	0.304	<0.001	<0.001	1.000
Artifact	5 (4–5)	5 (4–5)	4 (4–5)	<0.001	0.207	0.250	<0.001	0.023
Liver lesion/structure conspicuity	3 (3–3)	3 (3–4)	4 (4–4)	<0.001	0.615	0.003	<0.001	<0.001
Pancreas lesion/structure conspicuity	3 (3–4)	4 (4–4)	5 (4–5)	<0.001	0.497	0.001	<0.001	<0.001
Kidney lesion/structure conspicuity	4 (3–4)	5 (4–5)	5 (5–5)	<0.001	0.539	<0.001	<0.001	0.023
Vascular conspicuity	4 (3–4)	4 (4–4)	5 (4–5)	<0.001	0.611	<0.001	<0.001	<0.001
Overall image quality	3 (3–3)	3 (3–4)	4 (4–5)	<0.001	0.627	0.005	<0.001	<0.001

Note: Data are presented as median (interquartile range). [a]Bonferroni-adjusted P-values for three post-hoc Wilcoxon signed-rank tests within each row. [b]Direction was determined using the 1-sample Wilcoxon signed-rank test, indicating higher scores for Mixed image over the comparator.

Table S5. Qualitative scores on CT images (Reviewer 1) - Portal venous phase

Criterion	Mixed	VMI	DLR-VMI	Friedman p	Kendall W	Mixed vs VMI[a]	Mixed vs DLR[a]	VMI vs DLR[a]

Noise	4 (4–5)	3 (3–3)	5 (4–5)	<0.001	0.795	<0.001	0.009	<0.001
Contrast	2 (2–3)	4 (3–4)	5 (5–5)	<0.001	0.971	<0.001	<0.001	<0.001
Texture	5 (5–5)	5 (5–5)	4 (4–4)	<0.001	0.576	0.024[b]	<0.001	<0.001
Sharpness	5 (4–5)	4 (4–5)	4 (4–5)	<0.001	0.184	0.003	0.043	0.590
Artifact	5 (5–5)	5 (4–5)	5 (4–5)	0.037	0.077	1.000	0.059	0.102
Liver lesion/structure conspicuity	3 (3–4)	4 (4–4)	5 (4–5)	<0.001	0.613	<0.001	<0.001	<0.001
Pancreas lesion/structure conspicuity	3 (3–4)	4 (4–5)	5 (5–5)	<0.001	0.641	<0.001	<0.001	0.002
Kidney lesion/structure conspicuity	4 (4–4)	5 (4–5)	5 (5–5)	<0.001	0.542	<0.001	<0.001	0.020
Vascular conspicuity	3 (3–3)	4 (3–4)	5 (4–5)	<0.001	0.765	<0.001	<0.001	<0.001
Overall image quality	3 (2–3)	4 (3–4)	5 (4–5)	<0.001	0.863	<0.001	<0.001	<0.001

Note: Data are presented as median (interquartile range). [a]Bonferroni-adjusted P-values for three post-hoc Wilcoxon signed-rank tests within each row. [b]Direction was determined using the 1-sample Wilcoxon signed-rank test, indicating higher scores for Mixed image over the comparator.

Table S6. Qualitative scores on CT images (Reviewer 2) - Arterial phase

Criterion	Mixed	VMI	DLR-VMI	Friedman p	Kendall W	Mixed vs VMI[a]	Mixed vs DLR[a]	VMI vs DLR[a]
Noise	4 (4–5)	3 (2–3)	4 (4–4)	<0.001	0.702	<0.001	0.035	<0.001
Contrast	3 (2–3)	3 (3–4)	4 (4–4)	<0.001	0.579	0.002	<0.001	<0.001
Texture	4 (4–4)	4 (4–4)	4 (3–4)	0.003	0.137	1.000	0.009	0.021
Sharpness	4 (4–4)	4 (3–4)	4 (3–4)	0.028	0.083	0.053	0.015	1.000
Artifact	4 (4–5)	4 (4–4)	4 (4–4)	0.003	0.137	0.174	<0.001	0.337
Liver lesion/structure conspicuity	3 (3–3)	3 (3–4)	4 (3–4)	<0.001	0.193	0.314	<0.001	0.022
Pancreas lesion/structure conspicuity	3 (3–4)	4 (3–4)	4 (4–4)	<0.001	0.212	1.000	<0.001	0.003
Kidney lesion/structure conspicuity	4 (4–4)	4 (4–4)	4 (4–4)	0.014	0.099	1.000	0.043[b]	0.014[b]
Vascular conspicuity	3 (3–4)	3 (3–4)	4 (4–4)	<0.001	0.227	0.933	<0.001	0.001
Overall image quality	3 (3–4)	3 (3–3)	4 (3–4)	<0.001	0.272	1.000	0.001	<0.001

Note: Data are presented as median (interquartile range). [a]Bonferroni-adjusted P-values for three post-hoc Wilcoxon signed-rank tests within each row. [b]Direction was determined using the 1-sample Wilcoxon signed-rank test, indicating higher scores for DLR-VMI over the comparator.

Table S7. Qualitative scores on CT images (Reviewer 2) - Portal venous phase

Criterion	Mixed	VMI	DLR-VMI	Friedman p	Kendall W	Mixed vs VMI[a]	Mixed vs DLR[a]	VMI vs DLR-VMI[a]
Noise	4 (4–5)	3 (2–3)	4 (4–4)	<0.001	0.763	<0.001	0.221	<0.001
Contrast	2 (2–3)	3 (3–4)	4 (4–4)	<0.001	0.773	<0.001	<0.001	<0.001
Texture	4 (4–4)	4 (4–5)	4 (3–4)	<0.001	0.207	0.264	0.016	0.002
Sharpness	4 (4–4)	4 (4–4)	4 (4–4)	0.555	0.014	1.000	1.000	1.000
Artifact	4 (4–5)	4 (4–4)	4 (4–4)	0.493	0.016	1.000	0.779	1.000
Liver lesion/structure conspicuity	3 (2–3)	4 (3–4)	4 (4–4)	<0.001	0.398	0.011	<0.001	<0.001
Pancreas lesion/structure conspicuity	3 (3–4)	4 (3–4)	4 (4–4)	<0.001	0.370	0.032	<0.001	0.003
Kidney lesion/structure conspicuity	3 (3–4)	4 (4–4)	4 (4–4)	<0.001	0.336	0.005	<0.001	0.098
Vascular conspicuity	3 (2–3)	4 (3–4)	4 (4–4)	<0.001	0.427	0.003	<0.001	0.009
Overall image quality	3 (3–4)	3 (3–3)	4 (4–4)	<0.001	0.516	1.000	<0.001	<0.001

Note: Data are presented as median (interquartile range). [a]Bonferroni-adjusted P-values for three post-hoc Wilcoxon signed-rank tests within each row.

Table S8. Qualitative scores on IMD images (Reviewer 1) - Arterial phase

**Criterion**	**Conventional**	**DLR**	**Z[a]**	**Wilcoxon p**
Noise	2 (2–2)	3 (3–4)	5.391	<0.001
Contrast	2 (2–3)	4 (3–4)	5.369	<0.001
Texture	5 (5–5)	4 (4–5)	-3.513	<0.001
Sharpness	4 (3–4)	4 (4–5)	4.627	<0.001
Artifact	4 (4–5)	4 (4–5)	-1.129	0.259
Liver lesion/structure conspicuity	2 (2–3)	4 (3–4)	5.660	<0.001
Pancreas lesion/structure conspicuity	2 (2–3)	4 (3–5)	5.476	<0.001
Kidney lesion/structure conspicuity	4 (3–4)	5 (5–5)	5.313	<0.001

Vascular conspicuity	4 (3–4)	5 (4–5)	4.493	<0.001
Overall image quality	2 (2–3)	4 (3–4)	5.670	<0.001

Note: Data are presented as median (interquartile range). [a]Z was reported with the sign oriented such that positive Z indicates higher scores for DLR IMD image (negative Z indicates higher scores for the conventional IMD image).

Table S9. Qualitative scores on IMD images (Reviewer 1) - Portal venous phase

Criterion	Conventional	DLR	Z[a]	Wilcoxon p
Noise	2 (2–2)	4 (3–4)	5.698	<0.001
Contrast	2 (2–3)	5 (4–5)	5.731	<0.001
Texture	5 (5–5)	4 (4–5)	-3.962	<0.001
Sharpness	4 (3–4)	5 (4–5)	4.734	<0.001
Artifact	5 (4–5)	4 (4–5)	-1.706	0.088
Liver lesion/structure conspicuity	3 (2–3)	4 (4–5)	5.498	<0.001
Pancreas lesion/structure conspicuity	2 (2–3)	4 (4–5)	5.685	<0.001
Kidney lesion/structure conspicuity	4 (3–5)	5 (5–5)	4.944	<0.001
Vascular conspicuity	3 (2–3)	4 (4–5)	5.165	<0.001
Overall image quality	2 (2–3)	4 (4–5)	5.810	<0.001

Note: Data are presented as median (interquartile range). [a]Z was reported with the sign oriented such that positive Z indicates higher scores for DLR IMD image (negative Z indicates higher scores for the conventional IMD image).

Table S10. Qualitative scores on IMD images (Reviewer 2) - Arterial phase

Criterion	Conventional	DLR	Z[a]	Wilcoxon p
Noise	2 (2–2)	3 (3–4)	5.421	<0.001
Contrast	3 (2–3)	3 (3–4)	4.570	<0.001
Texture	4 (4–4)	4 (3–4)	-2.448	0.014
Sharpness	4 (3–4)	4 (3–4)	1.591	0.112
Artifact	4 (4–5)	4 (4–4)	-0.987	0.324
Liver lesion/structure conspicuity	2 (2–3)	3 (2–4)	4.878	<0.001
Pancreas lesion/structure conspicuity	2 (2–2)	3 (3–4)	5.488	<0.001

Kidney lesion/structure conspicuity	3 (3–4)	4 (4–4)	5.014	<0.001
Vascular conspicuity	3 (3–3)	4 (3–4)	4.288	<0.001
Overall image quality	2 (2–3)	3 (3–4)	4.876	<0.001

Note: Data are presented as median (interquartile range). [a]Z was reported with the sign oriented such that positive Z indicates higher scores for DLR IMD image (negative Z indicates higher scores for the conventional IMD image).

Table S11. Qualitative scores on IMD images (Reviewer 2) - Portal venous phase

Criterion	Conventional	DLR	Z[a]	Wilcoxon p[a]
Noise	2 (2–2)	3 (3–4)	5.392	<0.001
Contrast	3 (2–3)	4 (4–4)	5.475	<0.001
Texture	4 (4–5)	4 (4–4)	-2.882	0.004
Sharpness	4 (3–4)	4 (4–4)	2.537	0.011
Artifact	4 (4–5)	4 (4–4)	-2.030	0.042
Liver lesion/structure conspicuity	2 (2–3)	4 (3–4)	5.219	<0.001
Pancreas lesion/structure conspicuity	2 (2–3)	4 (3–4)	5.235	<0.001
Kidney lesion/structure conspicuity	3 (3–4)	4 (4–4)	4.811	<0.001
Vascular conspicuity	3 (2–3)	4 (3–4)	5.337	<0.001
Overall image quality	3 (2–3)	4 (4–4)	5.695	<0.001

Note: Data are presented as median (interquartile range). [a]Z was reported with the sign oriented such that positive Z indicates higher scores for DLR IMD image (negative Z indicates higher scores for the conventional IMD image).

Table S12. Inter-reader agreement (quadratic weighted kappa, κw)

Item	Mixed, VMI images– arterial phase (N=129)	Mixed, VMI images – portal venous phase (N=129)	IMD image – arterial phase (N=86)	IMD image – portal venous phase (N=86)
Noise	0.65	0.66	0.78	0.80
Contrast	0.63	0.79	0.69	0.71
Texture	0.34	0.34	0.48	0.45
Sharpness	0.41	0.27	0.55	0.57
Artifact	0.48	0.38	0.73	0.73

Liver conspicuity	0.53	0.65	0.74	0.72
Pancreas conspicuity	0.55	0.62	0.77	0.77
Kidney conspicuity	0.28	0.43	0.50	0.50
Vascular conspicuity	0.35	0.66	0.45	0.68
Overall quality	0.54	0.63	0.72	0.75

Supplementary Figure Legends

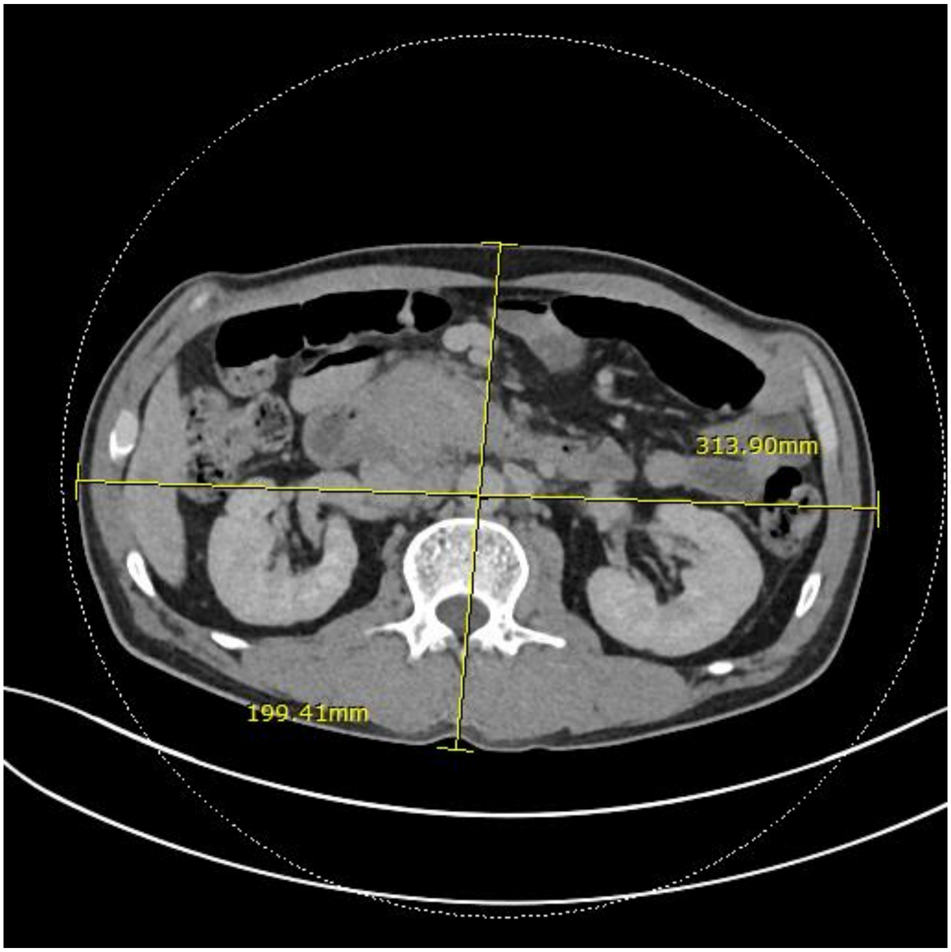

Figure S1: Size-specific dose estimate (SSDE) calculation in a 45-year-old man. An example of how to obtain the anteroposterior and lateral dimensions at the mid-scan image to calculate the effective diameter.

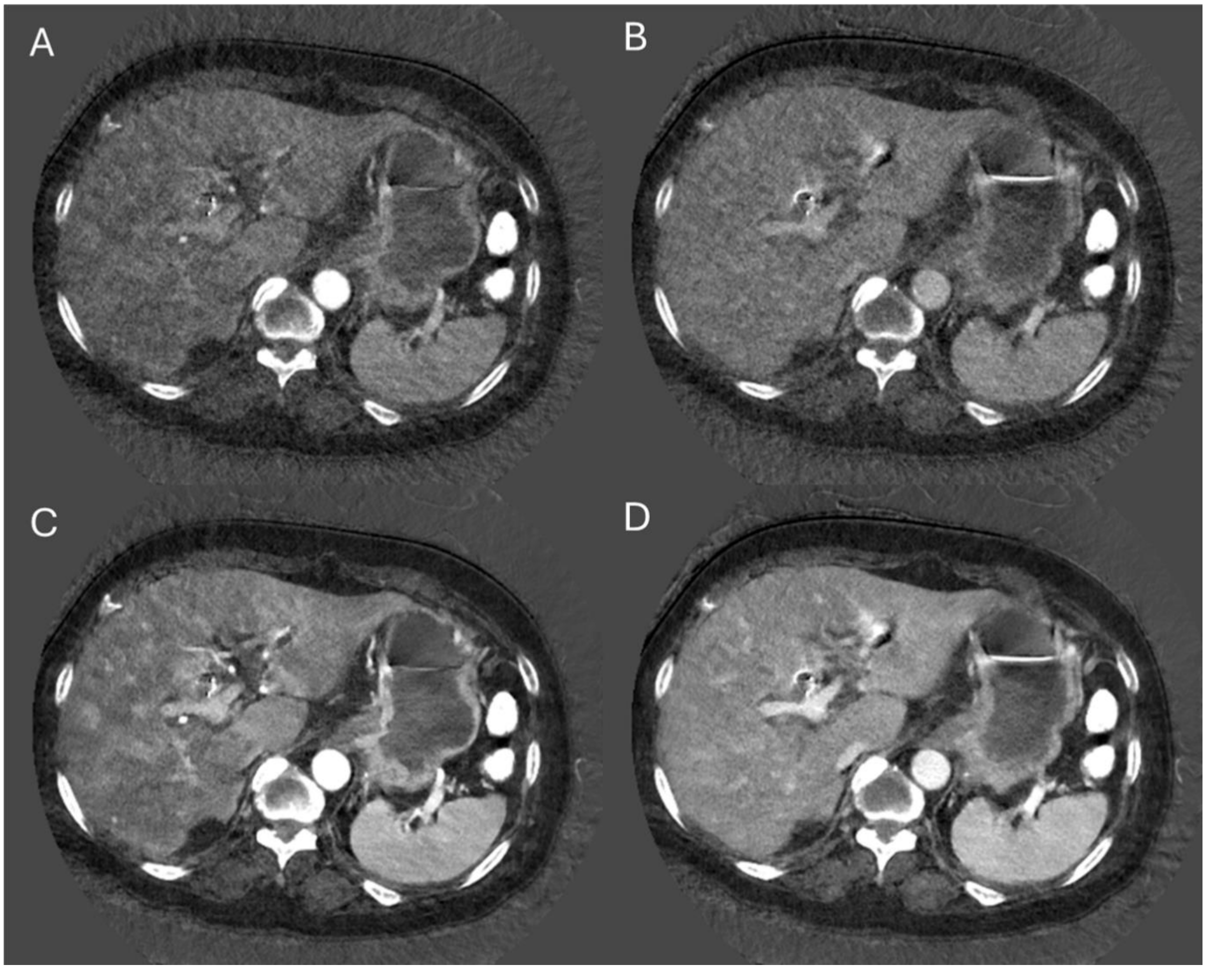

Figure S2: A 77-year-old woman with pancreatic cancer, status post ERPD, ERBD, and EUS-guided cholecystoduodenostomy. (A) Conventional IMD image, arterial phase. (B) Conventional IMD image, portal venous phase. (C) DLR-applied IMD image, arterial phase. (D) DLR-applied IMD image, portal venous phase. Using a five-point Likert scale (higher scores indicate better quality), overall image quality scores (Reviewer 1/Reviewer 2) were 2/2 (A), 3/2 (B), 4/4 (C), and 5/4 (D), demonstrating higher overall image quality scores for DLR-applied IMD images than for conventional IMD images in both phases. Both reviewers consistently reported improvements in noise and liver/pancreas conspicuity during the arterial phase, and in noise, contrast, sharpness, and liver/pancreas conspicuity during the portal venous phase. Liver SNRs are provided for each panel in the order displayed (A–D): SNRs of 1.81/4.55/3.34/11.60.
Abbreviations: DLR, deep learning-based reconstruction; ERBD, endoscopic retrograde biliary drainage; ERPD, endoscopic retrograde pancreatic drainage; EUS, endoscopic ultrasound; IMD, iodine material density; SNR, signal-to-noise ratio.
